# New Insights into Molecular Oncogenesis and Therapy of Uveal Melanoma

**DOI:** 10.3390/cancers11050694

**Published:** 2019-05-19

**Authors:** Sara Silvia Violanti, Ilaria Bononi, Carla Enrica Gallenga, Fernanda Martini, Mauro Tognon, Paolo Perri

**Affiliations:** 1 Department of Biomedical Sciences and Specialized Surgeries, School of Medicine, University of Ferrara and Eye Unit of University Hospital of Ferrara, 44124 Ferrara, Italy; sarasilvia.violanti@unife.it (S.S.V.); gllcln@unife.it (C.E.G.); 2 Department of Morphology, Surgery and Experimental Medicine, Section of Pathology, Oncology and Experimental Biology, School of Medicine, University of Ferrara, 44121 Ferrara, Italy; ilaria.bononi@unife.it (I.B.); mrf@unife.it (F.M.)

**Keywords:** uveal melanoma, gene, mutation, dysregulation, tumour virus

## Abstract

Uveal melanoma (UM), which is the most common cancer of the eye, was investigated in recent years by many teams in the field of biomedical sciences and eye clinicians. New knowledge was acquired on molecular pathways found to be dysregulated during the multistep process of oncogenesis, whereas novel therapeutic approaches gave significant results in the clinical applications. Uveal melanoma-affected patients greatly benefited from recent advances of the research in this eye cancer. Tumour biology, genetics, epigenetics and immunology contributed significantly in elucidating the role of different genes and related pathways during uveal melanoma onset/progression and UM treatments. Indeed, these investigations allowed identification of new target genes and to develop new therapeutic strategies/compounds to cure this aggressive melanoma of the eye. Unfortunately, the advances reported in the treatment of cutaneous melanoma have not produced analogous benefits in metastatic uveal melanoma. Nowadays, no systemic adjuvant therapy has been shown to improve overall survival or reduce the risk of metastasis. However, the increasing knowledge of this disease, and the encouraging results seen in clinical trials, offer promise for future effective therapies. Herein, different pathways/genes involved in uveal melanoma onset/progression were taken into consideration, together with novel therapeutic approaches.

## 1. Introduction

Uveal melanoma (UM) is a rare malignancy of the eye. However, it is the most common primary intraocular cancer in adults. Despite significant improvement in local tumour control rates, approaching 90% over a five-year period, patient survival has remained poor [1,2,3]. Metastasis occurs in up to 50% of patients, but less than 4% of them have detectable metastases at the time of diagnosis [4,5,6]. Many patients may have clinically-undetectable micro-metastasis at time of diagnosis, so the current trend is to consider uveal melanoma as a systemic disease [7,8,9]. Deeper knowledge of the molecular genetics, cell biology and immunology of UM has provided new insights into the molecular pathogenesis of this rare tumour. Currently, important research efforts are being addressed to develop effective adjuvant therapies in order to prevent metastatic disease, which may offer prophylactic systemic treatment. Thus far, no systemic adjuvant therapy has been shown to improve overall survival (OS) or reduce the risk of metastasis. Herein, we present an update on recent discoveries and results regarding the biological molecules underlying disease pathogenies and dissemination, as well as the development of tailored therapies.

## 2. Uveal Melanoma Genetics

### 2.1. Cytogenetics

Despite progress in diagnosis, allowing early detection even with small lesions and recent improvements in treatment, uveal melanomas remain a life-threatening disease showing a 50% metastatic rate and a mortality rate of 31% at five years, progressively increasing to 52% at 35 years [5,10]. Therefore, genetic analysis is useful in metastatic risk prediction, management and follow-up of patients with this malignancy. Cytogenetic abnormalities involving UM development include alterations to chromosomes 1, 3, 6 and 8 [11]. Monosomy of chromosome 3 is the most frequent karyotypic aberration, involving 50–60% of cases. It is the most frequently reported chromosomal alteration associated with poor prognosis [12,13,14], due to a strong relation with malignant tumour characteristics and histopathological factors, such as epithelioid cell type, closed microvascular loops, ciliary body involvement, larger tumour base diameter and thickness and cancer-related death from metastasis [15,16,17,18]. Usually, loss of or deletions to chromosome 3 are accompanied by amplification of chromosome 8 long arm, leading to a higher risk of metastasis [19,20] as well as a reported 10-year melanoma-related mortality rate of 71% [21].

**Figure 1:**
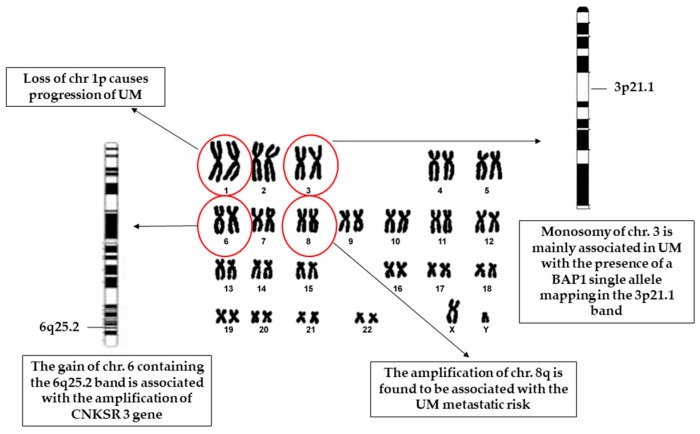
Schematic representation of the human karyotype showing the main chromosome aberrations/mutations, within red circles, detected in uveal melanoma (UM) cases. Karyograms of chromosomes 3 and 6 are represented with specific bands, 3p21.1 and 6q25.2, which contain BAP1 (*BRCA1*-associated protein 1), and *CNKSR 3* (member 3 of *CNKSR*: Connector enhancer of kinase suppressor of RAS), genes, respectively.

In 2014, van Beek J. G. et al. evaluated the prognostic value of an extraocular extension of uveal melanoma related to chromosomal abnormalities, concluding that gaining 8q and extraocular extensions are related to a worsened prognosis, while the addition of chromosome 3 loss leads to a significant reduction in metastasis-free survival [22]. Monosomy of chromosome 3 is the principal cytogenetic factor in metastatic disease. Furthermore, it has been demonstrated that there is frequent association in UM between somatic mutations of the tumour suppressor gene *BAP1* (3p21.1) and the single allele in chromosome 3, in cases of its monosomy [23]. Other less frequent abnormalities detected in uveal melanoma are: (i) loss of chromosome 1p, observed in association with monosomy 3 [24]; (ii) gain of chromosome 6, which is found to be the only “protective” cytogenetic alteration associated with good prognosis and non-metastatic disease [25,26,27]. A recent study showed that amplification of *CNKSR 3* (member 3 of *CNKSR*: Connector enhancer of kinase suppressor of *RAS*), membrane-associated guanylate kinase interacting protein-like gene, which maps on chromosome 6q25.2, extended metastatis-free survival in a group of uveal melanomas with monosomy of chromosome 3 [28], Figure 1.

### 2.2. Gene Mutations

Different studies have identified genes involved in uveal melanoma development, such as *GNAQ* (G protein subunit alpha q), *GNA11* (G protein subunit alpha 11), *CYSLTR2* (cysteinyl leukotriene receptor 2), *PLCB4* (phospholipase C, β4), *BAP1* (*BRCA1*-associated protein 1), *SF3B1* (splicing factor 3B subunit 1), *SRSF2* (serine and arginine rich splicing factor 2), *EIF1AX* (X-linked eukaryotic translation initiation factor 1A) and *TERT* (telomerase reverse transcriptase). These investigations have indicated that UM is characterised by a limited number of gene recurring mutations [29,30].

#### 2.2.1. *GNAQ/GNA11*

*GNAQ* and *GNA11* are two genes encoding for the α subunit of G protein-coupled receptors, which are responsible for extracellular signal transduction and intracellular pathways activation. Mutations in these genes are found in up to 91% of UM patients; therefore, being considered the principal driver of carcinogenesis [31]. Interestingly, they are mutually exclusive with a frequency of 42.2% and 32.6% for *GNAQ* and *GNA11*, respectively [32]. These mutations occur in the alpha subunits of G protein-coupled receptor (*GPCR*) as single amino acid substitutions at residues Gln209 (Q209, where glutamine is mutated to either leucine or proline) or, less frequently, at Arg183 (R183). The substitution of this critical glutamine determines constitutive GTPase activity of oncogenic Gq/11 subunits [31,33]. Somatic mutations activating these oncogenes trigger *MEK* (mitogen-activated kinase), protein kinase C and YAP (yes-associated protein) related pathways [34,35]. YAP is the major effect of the HIPPO pathway. When activated, the *YAP* gene product acts as an oncoprotein, promoting cell proliferation through nuclear transcription factors. *GNAQ* and *GNA11* mutations promote YAP activation and its nuclear localisation [36,37,38]. These data indicate that YAP could be considered a novel therapeutic target.

#### 2.2.2. *CYSLTR2*

Cysteinyl leukotriene receptor 2 (*CYSLTR2*) encodes a G-protein–coupled receptor, CysLT_2_R, involved in leukotriene-mediated signalling in inflammation and fibrosis. Moore and colleagues [39,40], analysing whole-genome and whole-exome sequencing data from 136 patients with uveal melanoma from multiple cohorts, found a previously undescribed mutation in *CYSLTR2* encoding a p.Leu129Gln substitution. This mutation was founded only in samples lacking mutations in *GNAQ*, *GNA11* and *PLCB4* (four of nine samples), suggesting that these mutations activate the same pathway. These findings reveal an oncogenic role for *CYSLTR2* in uveal melanoma through activation of Gα_q_ signalling, and further suggest that Leu129Gln CysLT_2_R may be a potential therapeutic target in UM.

#### 2.2.3. *PLCB4*

Phospholipase C β isoform 4 (*PLCB4*) have been investigated regarding their roles in the metabolism of inositol lipids and cancer. Johansson and colleagues [41] found a recurrent mutation in *PLCB4* (c.G1888T, p.D630Y, NM_000933), which was validated using Sanger sequencing. The identical mutation was also found in published UM sequence data (one of 56 tumours), supporting its role as a novel driver mutation in UM. *PLCB4* p.D630Y mutations are mutually exclusive with mutations in *GNA11* and *GNAQ*, consistent with *PLCB4* being the canonical downstream target of the former gene products. Taken together, these data suggest that the *PLCB4* hotspot mutation is similarly a gain-of-function mutation leading to activation of the same signalling pathway, promoting UM tumorigenesis.

#### 2.2.4. *BAP1*

The *BRCA-1* associated protein 1 (*BAP1*) gene, located on chromosome 3p21, encodes a nuclear ubiquitin carboxyl-terminal hydrolase enzyme that shows deubiquitinase activity. Nuclear localisation and deubiquitylation activity are necessary to carry out its tumour suppressor functions [42]. *BAP1* N-terminal domain mutations cause loss of protein expression and, consequently, loss of functions in this protein, which is normally involved in different cellular processes, such as cell cycle progression, DNA damage response and replication, stem cell pluripotency, histone modification and myeloid transformation [43,44]. It has been demonstrated that BAP1 is localised into the cytosol, at the endoplasmic reticulum (ER), where it can bind deubiquitylates, and stabilizes type-3 inositol-1,4,5-trisphosphate-receptor (*IP3R3*). Here, it can promote apoptosis, modulating the release of calcium (Ca2+) from the ER into the cytosol and mitochondria. Decreased levels of *BAP1* determine reduction of Ca2+ flux and IP3R3 levels, preventing apoptosis in mutated cells with a higher rate of cellular transformation [45,46]. Thus, *BAP1* depletion implicates a loss of cell differentiation and a gain in stem cell characteristics [47]. *BAP1* somatic mutations lead to development of many types of cancer, including lung adenocarcinoma, breast cancer, meningioma, malignant pleural mesothelioma, renal cell carcinoma and UM [48,49]. In uveal melanoma, *BAP1* mutations cause cell phenotype modifications, which are associated with metastatic disease in 84% of patients [23,47] and class 2 genetic features (with high metastatic potential). Onken et al. demonstrated that BAP1 depletion increases the amount of transmigration in uveal melanoma cells, giving valuable insight into the metastasization promoting mechanism [50].

Germline mutations in *BAP1* have been observed in 22% of familial uveal melanoma [51] and could be associated with early onset [52]. Therefore, *BAP1* screening could be a useful approach for identifying uveal melanoma predisposition, whereas the immunohistochemistry technique has been used as a solid method for prognostic purposes [53].

#### 2.2.5. *SF3B1*

Mutation at codon 625 of the splicing factor 3b subunit 1 (*SF3B1)* gene (chromosome 2q) is associated with uveal melanoma development. *SF3B1* is involved in mRNA splicing; therefore, its mutation causes splicing dysregulations and alters the transcription process [54]. *SF3B1* mutation has been found in about 15% to 19% of UM cases [55]. Interestingly, mutation in *SF3B1* gene is mutually exclusive of BAP1 mutations and is usually associated with disomy of chromosome 3 [56]. Despite *SF3B1* being associated with good prognosis and low metastatic potential (class 1 tumour), this subset of this tumour of the eye shows an intermediate risk of late onset metastasis, as confirmed by a large uveal melanoma disomy 3 cohort study recently conducted by Yavuzyigitoglu et al. using whole-exome sequencing analysis [57].

#### 2.2.6. *EIF1AX*

The eukaryotic translation initiator factor 1A X-linked (*EIF1AX*) gene (chromosome Xp) functions to initiate the translation process. It stimulates the transfer of Met-tRNAi (methionyl initiator tRNA) to the ribosomal subunit. Alterations in *EIF1AX* gene are usually missense involving the N-terminal portion of the encoded protein. The exact molecular mechanism that leads to uveal melanoma development is not fully understood. Recently, Johnson et al. suggested that an abnormal translation process might be responsible for a clonal selective advantage in cells displaying this alteration [58]. *EIF1AX* mutation, which is almost mutually exclusive with *BAP1* and *SF3B1* mutations, was found to be associated with the disomy of chromosome 3. Ewens et al. reported a 10-fold lower metastatic risk in patients with disomy 3 and *EIF1AX* mutation [59]. Thus, this mutation seems to be associated with good prognosis (class 1 tumour). The reported frequency of *EIF1AX* gene mutation is in the range of 8–18.9% of primary uveal melanoma cases [29,32].

#### 2.2.7. *TERT*

Telomerase reverse transcriptase (*TERT*) promoter mutation rarely causes uveal melanoma, representing about 1% of cases, while it is more common in conjunctival melanoma (41%) and primary-acquired melanosis (PAM) with atypia (8%). To the best of our knowledge, two cases of uveal melanoma were reported in literature due to *TERT* promoter mutation with increased expression of the gene in the tumour. Alteration in the *TERT* promoter gene, with an increase in its expression, leads to the immortalization of somatic cells. This mechanism is crucial to neoplastic conversion, because in normal cells telomere shortening narrows its replicative potential. Tumours carrying this mutation also showed *GNA11* gene alteration [32,60], Table 1.

## 3. Genetic Prognostic Tests

Since the first demonstration of correlation between cytogenetic changes and prognosis of uveal melanoma, shown by Prescher et al. [61] about the monosomy of chromosome 3, as a predictor of metastasis-free survival, several other prognostication methods, classifications and tests have been developed. Among the most used prognostication approach, fluorescence in situ hybridization (FISH), microsatelliteanalysis (MSA), multiplex ligation-dependent probe amplification (MLPA), single nucleotide polymorphism (SNP) array, and gene expression profiling (GEP) can be included [62]. Karyotyping via fluorescence in situ hybridization (FISH), may be one of the most used genetic methods for studying uveal melanoma. This approach is based on oligonucleotide probes to target specific chromosomal targets in a DNA sample. If the probe finds its target, the hybridization that occurred is detected by a microscopy. Even if several studies reported FISH as an accurate technique, other data demonstrate a variability failure rate, probably linked to subjectivity in analysing the results [63,64,65]. Therefore, in 2008 the MLPA-based test was firstly used on an uveal melanoma specimen [66]. This method also uses oligonucleotide probes to identify specific targets in specimens of DNA, but hybridization is identified by PCR through amplification of the amount of DNA. This faster and less-expensive approach with increased sensitivity can detect 31 loci across chromosomes 1p, 3, 6, allowing the prediction of high- and low-risk tumours [21,25,67,68]. When not enough sample is available for MLPA, an alternative genetic analysis test is represent by Microsatellite analysis (MSA), combining fluorescent probes with PCR [69,70,71]. This technique has shown accurate results for the identification of abnormalities in chromosome 3, but not for those in chromosomes 8 and 6 [70,72].

A novel technique, relying on gene expression profiling (GEP), that evaluates mRNA expression of different genes, enabled uveal melanoma to be stratified into two prognostic groups: (i) class 1 tumour, with low metastasis risk and a 95% eight-year survival rate; (ii) class 2 tumour, with high metastatis risk and a 31% eight-year survival rate [73]. The analysis of these two subgroups highlighted that: (i) class 1 tumour is characterised by a chromosome 6p gain; (ii) class 2 tumour is characterised by a loss of chromosome 3, associated with a gain of chromosome 8q. In addition, Field et al. clustered class 1 tumour into 1A and 1B, showing a 2% and 21% five-year metastatic risk, respectively [33]. Likewise, Onken et al. divided class 2 tumour into 2A and 2B, the latter characterised by a chromosome 8p loss with more aggressive behaviour resulting in early metastatic disease [74].

Recently, the cancer-testis antigen PRAME (preferentially expressed antigen in melanoma) has been evaluated as an independent biomarker to predict metastasis risk both in class 1 and class 2 tumours [75,76]. The authors have established a threshold for PRAME mRNA expression in order to define PRAME+ status. Interestingly, PRAME+ status was statistically associated with a larger tumour diameter after analysing the TCGA (The Cancer Genome Atlas Research Network dataset). This finding implies that advanced growth is necessary to activate PRAME transcription. As far as chromosomal alteration is concerned, PRAME+ tumours were strongly associated with 6p loss, 6q loss and 8p gain both in class 1 and class 2 tumours, while 8p loss was associated with PRAME+ status only in class 2 tumours. Interestingly, PRAME was not associated with monosomy 3, either in class 1 or class 2. Moreover, analysing uveal melanoma drivers of mutations, PRAME+ status was found to be directly associated with SF3B1 alterations, while it was inversely associated with EIF1AX changes in class 1 tumours. It has been reported that the PRAME promoter region became hypomethylated and thus activated during UM progression. These features confer a valid prognostic value to PRAME analysis [76]. In this context, it is worth citing from a recent review by Dogrusöz et al.: “genetic prognostication in UM is an advancing field in which continued research is expected to further enhance prognostic accuracy, improve patient counselling, planning of follow-up, and trial enrolment, and contribute to the identification of new therapeutic targets” [77].

## 4. UM Association with Small DNA Tumour Polyomaviruses BKPyV and SV40

Many factors are involved in the etiopathogenesis of UM, as with other human tumours, (see above). In the onset/progression of different cancers, including UM, many chromosome aberrations and point mutations may occur [29]. Environmental risk factors could act as mutagenic and oncogenic agents. Different biological organisms, such as tumorigenic viruses have been found to be associated with human tumours of different types [78,79]. Indeed, among polyomaviruses (PyVs) with oncogenic potential, simian virus 40 (SV40) and BK (BKPyV) have been detected in human tumours [80,81,82]. These PyVs exert their transforming capabilities in human cells through the products of their viral oncogenes, the large T antigen (Tag) and small t antigen (tag) [81]. Tag affects cellular growth–control mechanisms, whereas tag activates the Wnt pathway [83].

These activities adversely affect the cellular genome, which accumulates many gene mutations or chromosome aberrations. Then, in the absence of p53 functions, due to Tag binding, cellular DNA remains unrepaired, leading the genome to “derail” [84]. SV40/BKPyV and uveal melanoma association has been investigated using an immunological approach. Indeed, the recent development of specific, sensitive serologic tests for SV40 and BKPyV [85,86], which consist in indirect enzyme-linked immunosorbent assays (ELISAs) using synthetic peptides as mimotopes or antigens of their viral proteins (VPs), have enabled the presence of specific IgG antibodies to be investigated in sera from UM-affected patients. UM sera analysed by indirect ELISAs using two synthetic peptides corresponding to mimotopes of SV40/BKPyV viral capsid proteins showed a higher prevalence of SV40/BKPyV-antibodies compared to healthy subjects [78,87]. However, these studies, which assessed the association between UM and oncogenic polyomaviruses SV40 and BKPyV, are not proof of a causal relationship with UM onset/progression.

## 5. Current Therapeutic Options for Primary Tumours

Management of posterior uveal melanoma has been evolving over the past decade. In the past, enucleation was the most common treatment option. However, when Zimmerman and colleagues [88] proposed that enucleation might be correlated with an increased risk of metastatic disease, a debate on managing this malignancy, and increased interest in alternative treatments began to surface. Nowadays, the management of posterior uveal melanoma depends on tumour size, location, and extent, systemic status and visual acuity on presentation [89,90,91]. Briefly, it can be divided into eye-sparing treatment (including radiation, laser therapy, or surgery) or enucleation [92]. Currently, the radiation approach, with brachytherapy and proton beam irradiation, is the most commonly used treatment method. Several forms of radiotherapy have been developed to maximize the dose of radiation applied to the tumour, while reducing collateral damage to the surrounding tissues. The purpose of episcleral brachytherapy is to deliver radiation using different applicators (plaques) coated with silver, shaped in different forms and diameters, and containing a radioactive source. Traditionally, different types of radionuclides are used for ophthalmic brachytherapy, including ruthenium-106 (106Ru), iodine-125 (125I) and palladium-103 (103Pd). Ruthenium-106 was firstly introduced into ocular oncology by Lommatsch in the 1970s [93,94]. 106Ru is a beta radiation emitter suitable for treating small, circumscribed lesions, since the beta rays, which are emitted, have a limited range. This beta emitter is the principal radionuclide used in Europe for the treatment of uveal melanoma of up to 5 mm in thickness. Conversely, iodine plaques emitting gamma radiation are used to treat tumours with a thickness of 10 mm. However, these gamma emitter plaques have the disadvantage of delivering large doses of irradiation to healthy ocular structures [93,95,96]. The majority of primary uveal melanoma lesions in the U.S. are treated with plaque brachytherapy, which is supported by the results of the COMS (Collaborative Ocular Melanoma Study) trial [97,98,99]. This study was the largest one ever to be performed in ocular oncology, including 43 participating centres, which included more than 2000 patients. Participants were randomised with medium-sized choroidal melanomas to primary therapy with iodine-125 (125I) brachytherapy versus enucleation. Data demonstrated no difference in long-term survival rates between the two treated groups.

Proton beam therapy uses beams of protons (sub-atomic particles) as its ionizing radiation source. Linear accelerator or fixed radioactive sources can create multiple proton beams, which can be precisely directed to converge at a uveal melanoma irrespective of tumour size, shape or location. The total dose of radiation delivered (53 to 70 Gy) is usually administrated into several daily fractions, conventionally 4lxs to 10, to maximize the therapeutic index of treatment, with the hope of reducing damage to healthy tissues. Proton beam radiotherapy can be used for all types of uveal melanomas. In choroidal tumours, it is usually administered with safety margins of 2.0–2.5 mm; due to the tendency to grow large, anterior, iris tumours requiring a wider safety margin, circumferentially along the ciliary body. Even when plaque radiotherapy of uveal melanoma is possible, proton beam radiotherapy may be preferable since it reduces the chances of collateral damage to surrounding tissues. However, equipment for this treatment is only available in a small number of centres [93,100].

Although, excellent rates of local disease control can be achieved with surgery or radiotherapy, about 50% of patients with uveal melanoma will develop metastatic disease within 15 years of initial diagnosis [1,5,101,102]. Given the unique vasculature of the eye and the distinct biology of this tumour, the predilection site for metastatic spread is the liver [1,5,69,103]. Despite recent advances in understanding molecular characteristics having helped to determine which subtypes are most likely to progress, no therapy has been shown to improve overall survival for uveal melanoma in either adjuvant or metastatic settings [33,104,105]. Increasing knowledge of the molecular aspect of uveal melanoma has led to the development of new therapeutic approaches, and recently intensive activity has been focused on developing and trialling targeted systemic therapies. Continued advances in understanding of the molecular mechanisms of uveal melanoma will help prognostic markers, therapeutic targets and effective treatment approaches to be identified.

## 6. Current, Innovative Therapeutic Options for Metastatic Tumours

### 6.1. Liver-Directed Approach

Approximately 30%–50% of UM patients successfully treated for primary tumour develop metastases. The liver is the most common site of metastases, whereas approximately 50% of patients have isolated liver metastases [1,5,103]. An analysis of patients enrolled in the COMS study found that 93% of patients had liver metastases at time of death [106]. Of those who had only one site of metastasis, the liver was involved in 95% of cases and the median overall survival (OS) for patients with liver metastases was six to 12 months [1,5].

Several different systemic and regional treatment strategies have been explored, but survival rates have not improved [107]. Nowadays, a surgical approach is considered the gold standard for achieving a curative resection (R0 resection) for UM metastases, whereas chemotherapy is used for unresectable tumours [101]. Unfortunately, the number of patients with resectable liver metastasis is limited (<10%). Most liver metastases are multiple with both hepatic lobes involved [108]. Systemic chemotherapy, both single substance (Dacarbazine or Temozolomide [109]) or combination treatments (BOLD regimen [110], gemcitabine with treosulfan [111]) have shown response rates of less than 10%. In order to reduce systemic toxicity and side effects, alternative liver approaches have been developed. Both transarterial chemoembolization (TACE) with cisplatin and carboplatin and selective internal radiation therapy (SIRT) have shown a partial response (57% of patients with a median survival of nine months versus 62% of patients with a median survival of seven months, respectively) [112,113]. In a retrospective analysis on 3873 patients with uveal melanoma, Mariani et al. [114] reported on a median overall postoperative survival of 14 months for 255 patients who had undergone surgical resection. Notably, the median overall survival rate increased to 27 months in the selective cohort of 76 patients where a microscopically complete resection (R0) was obtained [114]. Isolated hepatic perfusion (IHP), whether open or percutaneous, has emerged as a viable treatment option [115]. These organ-specific approaches allow the liver to be perfused with high doses of cytotoxic chemotherapy, minimizing systemic exposure. Compared to systemic chemotherapy, hepatic intra-arterial chemotherapy (HIA) has shown a slightly higher overall response rate, but no increase in overall survival [116]. In patients with refractory uveal melanoma liver metastases, IHP, performed with alkylating agent melphalan or tumour necrosis factor α (TNFα), has been reported to achieve hepatic response rates of greater than 50%. The progression-free survival was 12 months, thus exceeding outcomes obtained with systemic chemotherapy [117]. The EORTC 18021 phase III trial (NCT00110123) [116] stated that despite improved anti-tumour efficacy enhancing rate response (RR) and improving progression free survival (PFS), IHP did not increase the OS of UM patients with liver metastases only. Leyvraz and co-workers compared IHP with fotemustine to intravenous systemic chemotherapy in treating liver metastasis from UM, demonstrating that IHP is more effective than systemic chemotherapy in controlling liver metastases. A median PFS of 4.5 months and response rate of 10.5% with IHP versus 3.7 months and 2.4% was obtained, respectively, with intravenous treatment [116].

A phase II follow-up trial confirmed a significant potential survival benefit of 14 months for patients treated with IHP compared to the longest surviving patients in Sweden during the same time period [118].

A randomised controlled multi-centre study (SCANDIUM) is currently recruiting patients with isolated liver metastases from uveal melanoma to evaluate if IHP may increase overall survival compared with best alternative care (BAC) (NCT01785316).

Percutaneous hepatic perfusion (PHP) is an alternative endovascular procedure with vascular isolation and perfusion of the liver. When compared to best alternative care, PHP has shown an improvement in overall rate response (ORR) but not in OS [115,116,117]. During PHP, chemotherapy is infused via a percutaneous catheter in the hepatic artery. The liver venous outflow is filtered through an extracorporeal filtration system using a double-balloon catheter positioned percutaneously in the retrohepatic inferior vena cava. Then, a catheter in the internal jugular vein re-transfuses the blood [117]. In the FOCUS study (NCT02678572) an ongoing multi-centre clinical trial is randomising patients with hepatic dominant metastatic ocular melanoma to either PHP with melphalan or one of the following alternative care options: transarterial chemoembolization, dacarbazine, ipilimumab or pembrolizumab. The estimated primary completion date is in 2020.

Recent investigations have focused on embolisation techniques, especially in association with systemic immune checkpoint inhibitors. It has been postulated that these procedures may lead to an increased release of tumour antigens to the immune system, and the concurrent use of immune checkpoint inhibitors may be helpful in controlling the disease [101,119]. Embolisation approaches include hepatic arterial chemoembolisation with different of agents (fotemustine, BCNU, cisplatin), bland embolisation, and radioembolisation with yttrium-90 (90Y)-labeled biocompatible microspheres injected into the hepatic artery. In 2011, Gonsalves et al. [120] show results of 32 patients UM and liver metastasis treated with radioembolization therapy, proposed as salvage therapy after failure of TACE or immunembolisation. Based on pre-treatment tumour burden, authors divided patients into three categories: <25% (*n* = 25), 25% to 50% (*n* = 5), and >50% (*n* = 2). At the end of the clinical follow-up, the overall survival for the entire cohort of patients ranged from 1.0 to 29.0 months (with a median of 10.0 months). A significantly longer median overall survival was found in the group with a pre-treatment tumour burden <25% (10.5 months; range 3.1–29.0 months) than in the other two groups (3.9 months; range 1.0–12.1 months). In term of treatment response, a complete response was observed in one patient. One patient had a partial response and 56.3% of patients (*n* = 18) had stable disease. In comparison to patients with progressive disease, those with stable disease or better, had a significantly longer overall survival (4.9 versus 14.7 months, respectively).

In a similar study with a smaller cohort of 13 patients, Klingenstein et al. showed benefit for patients with liver metastases treated with radioembolisation. In 77% of patients, there was partial remission or a stabilising of the disease was observed [112]. A clinical trial studying a combination of Y-labelled microspheres with sorafenib (NCT01893099) was concluded, but results have not yet been published. Immunoembolisation using granulocyte-macrophage colony-stimulating factor (GM-CSF), represents another approach, as does a randomised phase II study comparing immunembolisation (IE) and bland embolisation (BE). Statistically significant survival advantage was reported in the immunembolisation cohort of patients with at least 20% liver involvement. In contrast to the overall survival of 17.2 months obtained with BE, the IE group showed 21.5 months of OS [121].

### 6.2. Chemotherapy

Uveal melanoma tumours, both primary and metastatic, are essentially highly resistant to traditional chemotherapy [122]. Chemotherapy regimens utilising differing combinations, adopted to treat skin melanoma, such as cisplatin, fotemustine, dacarbazine, temozolomide and treosulfan, have shown unsatisfying results in uveal melanoma. Response rates range from 0% to 15%, and responders have a very limited prolonged survival [107,109,123,124,125,126]. Hence, there is currently active investigation into developing and trialling targeted and immune-therapies.

### 6.3. Targeted Therapy

Improved understanding of cutaneous and uveal melanoma mutations, particularly those involving the mitogen-activated protein kinase pathway have led to the development of targeted therapies. Targeted blockades of regulatory growth signalling pathways such as agents against components of the mitogen-activated protein kinase (MAPK) pathway, BRAF and NRAS have recently been introduced in the armamentarium for advanced cutaneous melanoma management. These agents significantly extended the survival of patients with cutaneous melanoma. Unfortunately, this success has not extended to UM [127,128,129,130]. Since primary uveal melanoma tumours and liver metastases, characterised by mutations in GNAQ or GNA11, have constitutive activation MAPK signalling via alternative, similar therapies targeting downstream effectors of the MEK pathway, Akt and protein kinase C (PKC) were recently investigated. Preclinical models have shown that UM cell lines are susceptible to the inhibition of the MAPK pathway by MEK inhibitors [131].

Selumetinib (AZD6244) is an oral, potent, highly selective non-ATP competitive inhibitor of MEK1/2 [132]. A randomised, phase II study in 101 patients with advanced uveal melanoma compared selumetinib versus chemotherapy (temozolomide or dacarbazine). Selumetinib resulted in a modest improved progression-free survival PFS (15.9 versus 7 weeks, *p* < 0.001) and response rate; however, no significant improvement in overall survival was reported (11.8 versus 9 months, *p* = 0.09) [132]. Subsequently, the SUMIT study (NCT01974752), a double-blind, placebo-controlled phase III trial was conducted, in which 129 patients with metastatic uveal melanoma with no prior systemic therapy were randomly assigned to selumetinib plus dacarbazine versus dacarbazine alone. Unfortunately, the primary endpoint, the median PFS, was not significantly improved in the selumetinib arm compared to dacarbazine alone (2.8 versus 1.8 months, *p* = 0.32). The objective response rate was 3% with the selumetinib cohort and 0% with dacarbazine cohort with no significant difference (*p* = 0.36) [133].

To further investigate the role and optimise the efficacy of the MEK inhibitor in UM, while minimizing toxicity and the effect of drug-related feedback reactivation, other studies are underway [134]. Selumetinib, with an intermittent dosing schedule is being tested in treatment-naïve metastatic uveal melanoma, as monotherapy in a phase Ib trial (NCT02768766). The randomised phase II SelPac trial investigated the activity of selumetinib in three treatment arms: continuous selumetinib, continuous and intermittent selumetinib in combination paclitaxel, based on evidence that the combination increases induction of apoptosis in preclinical models [135,136].

Mutations in GNAQ and GNA11 activate both MEK and Akt, thus an alternative approach might be offered by a simultaneous inhibition of these two pathways. Trametinib, another MEK inhibitor, was evaluated in a phase II trial, both as monotherapy and in combination with an Akt/PKB (protein kinase B) inhibitor, GSK2141785, in 40 patients with metastatic uveal melanoma. Unfortunately, no benefit to PFS was detected with the addition of Akt inhibition [137].

Constitutive protein kinase C (PKC) and phospholipase C β (PLCβ) activation has also been implicated in stimulations of the MAPK pathway in uveal melanoma. Investigation into agents targeting these components are also underway. A phase I study (NCT02601378) is recruiting patients to test the PKC inhibitor oral agent LXS196. Preliminary data shown during last Society of Melanoma Research (SRM) Congress [138] suggest encouraging clinical activity of LXS196 as monotherapy in patients with metastatic UM. In 50 evaluated patients, two had partial responses (PR), and 33 patients showned stable disease. In another phase I study on PKC inhibitor AEB071 (Sotrastaurin), partial responses were observed in only four of 153 patients (3%). In total, 76 patients (50%) had reached stable disease conditions, while the median PFS was 3.5 months [139]. Preclinical studies have documented the synergetic effect of PKC inhibitor AEB071 with PI3K inhibitor BYL719 in GNAQ- and GNA11-mutant cell lines, offering an alternative approach to target the signalling pathways [139]. The combination of AEB071 and BYL719 is being evaluating in a phase Ib trial in patients with metastatic uveal melanoma (NCT02273219).

Furthermore, as a consequence of GNAQ/GNA11 mutation, an upregulation of MET has been implicated in uveal melanoma. MET and VEGF signalling are implicated in angiogenesis, invasion and metastasis.

Cabozantinib is an orally bioavailable inhibitor of tyrosine kinases including, c-MET, VEGF and Axl that has been recently approved by the Food and Drug Administration (FDA) for treatment of patients with advanced renal cell carcinoma or medullary thyroid cancer. In preclinical studies, it has shown anti-metastatic activity in a xenograft mouse model of the metastatic uveal melanoma [139]. A phase II randomised discontinuation trial assessed cabozantinib (XL184), in a cohort of 23 patients with metastatic melanoma and reported that the median OS was 12.6 months and the median PFS was 4.8 months, thereby suggesting potential for clinical activity [139]. Based on these promising results, a randomised phase II study compared the MET inhibitor Cabozantinib to Temozolomide or Dacarbazine in patients with metastatic uveal melanoma (NCT01835145), but results are yet to be published. Recently, it has been demonstrated the role of cMET in the primary resistance to MEK inhibitors in metastatic UM with GNAQ/11 mutation. Even if the microenvironment mechanism regulating these responses is poorly understood, the combined inhibition of cMET and MEK may improve the responses to targeted therapies of metastatic UM. Although this preliminary research is promising, further studies are needed [140].

Sorafenib is another oral multikinase inhibitor with a potent activity against serine/threonine kinase isoforms, including RAF kinase, vascular endothelial growth factor receptor (VEGFR) and the platelet-derived growth factor receptor (PDGFR). A single arm phase II trial evaluated sorafenib as a single agent in patients with MUM and revealed stable disease at 24 weeks in 31.2% of patients [141]. Previously, a phase II study had evaluated the efficacy of sorafenib in combination with carboplatin and paclitaxel (CP) in metastatic uveal melanoma. Although minor tumour responses and stable disease were observed (tumour regression <30% and the four-month PFS) no significant efficacy occurred (ORR = 0% [95% CI: 0–14%]) [142].

Similar results indicating some level of anti-tumour activity were demonstrated in a randomised discontinuation, blinded, placebo-controlled phase II study (STREAM) (NCT01377025) [142]. A cohort of 149 patients with chemotherapy-naive metastatic UM were enrolled. Seventy-eight patients, that showed stable disease conditions, were then randomised to continuation or discontinuation of sorafenib. Significant improvement in PFS compared to placebo (5.5 versus 1.9 months, *p* = 0.0079) was associated with the continuation regimen. Notably, two patients (1.3%) achieved a partial response, suggesting some degree of clinical activity despite the overall low ORR. Although sorafenib seems to promote the stabilisation of the disease, it failed to improve OS of metastatic UM patients [142].

Interesting results were recently reported by Valsecchi et al. [121]. In this retrospective study, the use of low-dose sunitinib, a non-selective c-Kit inhibitor, in the adjuvant setting was associated with better overall survival. Investigators are currently validating the results of this study in a randomised, non-comparative (sunitinib and valproic acid) phase II clinical trial (NCT02068586) in patients with high-risk of uveal melanoma. Previously, the randomised multicentre phase II (SUAVE) trial failed to show any survival benefit with sunitinib compared to dacarbazine in 74 patients with untreated metastatic uveal melanoma [92].

### 6.4. Immunotherapy

#### 6.4.1. Checkpoint Inhibition

In recent years, immunotherapy have gained attention due to favourable results obtained with immunological checkpoint inhibitors targeting cytotoxic T lymphocyte- associated antigen 4 (CTLA-4), ipilimumab and programmed cell death-1 (PD-1) pembrolizumab, in the management of cutaneous melanoma [143,144]. The purpose of immunotherapy is to surround the tumour or metastases with an immunogenic environment, using the therapeutic potential immune system effector and tumour-specific antibodies. Unfortunately, similar benefits of cutaneous melanoma have not been observed in uveal melanoma. It has been postulated that UM, arising in an immune-privileged organ, may establish mechanisms to evade the immune system. Furthermore, the difference response to this approach may partially be due to the differences in overall tumour mutational burden. Compared to other cancers, uveal melanoma is characterised by a low mutational burden with few potential neo-epitopes for effective antitumour immunity [145,146,147]. Promising responses for metastatic UM have been observed in some retrospective or small prospective clinical trials that evaluated antibodies against two of the main molecules involved in the inhibition of the T-cell activation, CTLA-4 and PD-1.

#### 6.4.2. Anti-CTLA-4 Therapy

CTLA-4 is a negative regulator of T cells binding to CD80 or CD86 on antigen-presenting cells. Ipilimumab, a monoclonal antibody blocking the CTLA-4 receptor, previously approved by the FDA in 2011 as a second line for the treatment of advanced melanoma, is now used in adjuvant settings for treating different cancers such as lung cancer, metastatic renal carcinoma or Hodgkin lymphoma [148]. Even if the two randomised phase III studies have demonstrated that ipilimumab offers benefits in OS for patients with advanced melanoma, not including UM, there are some smaller prospective and retrospective studies that have reported limited clinical activity [143,144]. Luke and co-workers [149] in one of the larger retrospective, multicentre analyses, including 39 patients with metastatic uveal melanoma, provided evidence that ipilimumab has an immune-related response rate of ∼5%. The median OS from the first dose of ipilimumab was 9.6 months. However, other multiple phase II trials have shown low RRs ranging from 0% to 5%, a PFS of ~3 months, and an OS of less than one year [149,150,151,152,153]. The GEM-1 study [154] an open label, single arm phase II trial evaluated first line ipilimumab in 32 adult patients newly diagnosed with MUM and was conducted in five centres in Spain belonging to the Spanish Melanoma Group (GEM). Patients received four high doses of ipilimumab (10 mg/kg) every three weeks and a maintenance dose every 12 weeks. After a median follow-up of 5.5 months on thirteen evaluable patients, a partial response was documented in only one (7.7%) and a stable response in six (46.2%). Subsequently, Zimmer et al. [155] conducted the phase II DeCOG (Dermatologic Cooperative Oncology Group) trial on both pre-treated and treatment-naïve MUM patients (45 and eight patients, respectively) and reported median PFS of only 2.8 months and median OS of only 6.8 months. No partial or complete responses were observed [155].

Tremelimumab, another CTLA-4-blocking antibody, was evaluated by Joshua et al. [156] in a multicentre prospective phase II trial. Eleven patients were enrolled and received 15 mg/kg tremelimumab every 90 days for up to four cycles, but no responses were obtained among them. The median PFS and OS were 2.9 months (95% CI 2.8–3.0) 12.8 months (95% CI 3.8–19.7), respectively. Based on poor results and lack of efficacy, the trial was stopped at the first interim analysis.

### 6.5. Anti-PD-1 Therapy

Programmed cell death-1 (PD-1), a member of the CD28 superfamily, delivers negative signals upon interaction with its two ligands, PD-L1 or PD-L2. PD-1 exerts a wider range of immune-regulatory roles in T cells activation and tolerance, inhibiting their activity and proliferation in peripheral tissues. Agents targeting the PD-1/PD-L1 signalling have shown promising response in the treatments of many types of solid tumours both in clinical trials and settings. Nowadays, two antibodies against PD-1, nivolumab and pembrolizumab have been recently approved by FDA and/or European Medicines Agency (EMA) for treatment of advanced cutaneous melanoma. These molecules have demonstrated greater outcomes and an improved toxicity profile compared to ipilimumab [157,158,159]. Although the efficacy of these molecules has only been evaluated in retrospective case series on patients with advanced uveal melanoma, both activity and favourable toxicity profiles have been reported.

Kottschade et al. [160] investigated a cohort of 10 patients with metastatic UM treated with pembrolizumab, given at a dose of 2 mg/kg every three weeks for up to two years, through the expanded access program (EAP). One complete response, one stable disease condition (10%), and two partial responses (20%) were reported with an ORR of 30%. No OS data were reported and PFS was only 18 weeks (range 3.14–49.3).

Encouraging results were documented by Karydis and co-workers [161] in another retrospective case series. Of the 25 metastatic uveal melanoma patients enrolled and treated with pembrolizumab, two patients showed a partial response and six patients stable disease conditions. Similar results were reported in a large series of 58 patients with advance uveal melanoma who received PD-1 antibodies (pembrolizumab or nivolumab) or PD-L1 antibodies (atezolizumab) [162]. In this cohort, objective tumour responses were observed in two patients for an overall response rate of 3.6% (95% CI 1.8–22.5%), and stable disease conditions in five (9%). Median PFS and OS were 2.6 months (95% CI 2.4–2.8 months) and 7.6 months (95% CI 0.7–14.6 months), respectively.

The first efficacy and updated safety data from CheckMate 172 trials were encouraging. This phase II trial (NCT02156804) is the biggest one that has evaluated efficacy and safety for nivolumab monotherapy (3 mg/kg) in 734 patients with different melanoma subtypes, including UM, after anti-CTLA-4 therapy. At the end of a follow-up of ≥one year, out of 75 patients in the UM subgroup, 15 patients had disease stabilisation (44%) and two showed PR. The OS rate at one year was 47% and the median OS was 11 months.

The results of these retrospective series underline that durable, objective tumour responses and sustained disease control occurred, but were rare. Thus, nowadays PD-1 and PD-L1 antibodies are beneficial only in a small subset of metastatic uveal melanoma. As Javed et al. postulated [163], the lower PD-L1 expression in metastatic UM, compared to cutaneous melanoma, may suggest a rationale for failure of PD-1 and PD-L1 inhibitor agents, and an alternative mechanisms of immune evasion may occur. Further prospective studies are needed to confirm the low response rate.

### 6.6. Combined CTLA-4 and PD-1 Blockade

Since CTLA-4 and PD-1 suppress different phases of T cell activation, centrally and peripherally, respectively, it has been postulated that a combination of these therapies might be more effective. The CheckMate218 (NCT02186249) trial evaluated a combination of ipilimumab with nivolumab or pembrolizumab in 64 patients with metastatic melanoma, including six patients with uveal melanoma. Unfortunately, results from the UM subgroup have shown a median PFS of 2.8 months (95% CI 1.2–4.6 months) and an ORR of 0% (95% CI 0–51%) [161]. Currently, there are another two prospective phase II trials (NCT01585194, NCT02626962) investigating the combination of ipilimumab and nivolumab in metastatic UM. Since high mutation burden is predictive of response to immune checkpoint inhibitors, it has been proposed that the low mutation burden in patients with uveal melanoma may be related to the limited activity of PD1 suppressor. Recently, it has been reported few cases of UM with high tumour mutation burden correlated with a germline loss-of-function MBD4 mutation that were responsive to these agents. These findings can be helpful in stratification of the potential responsive UM to Checkpoint Inhibitor [164,165].

However, given the published data to date, overall activity of anti-CTLA-4 and anti-PD-1/PD-L1 agents in advanced uveal melanoma is limited and should not be used in frontline treatment as it stands in cutaneous melanoma.

### 6.7. Innovative Immune-Based Approaches

Despite the limited clinical activity obtained with immune checkpoint inhibition, other novel immune-based approaches showing promising results in treating this disease have recently been reported. Depending on these results, future scenarios could dramatically change. Thus far, different clinical trials have tested infusion autologous-modified dendritic cells. In a prospective study, 14 patients with metastatic uveal melanoma were vaccinated with autologous dendritic cells loaded with tumour peptides of gp100 and tyrosinase. Four of the vaccinated patients showed long overall survival, and the median OS was 19.2 months [166,167]. Currently, a phase III trial (NCT01983748) is recruiting patients suffering from uveal melanoma typed positive for monosomy 3 and without evidence for metastases who will be vaccinated over a two-year period with dendritic cells loaded with autologous tumour RNA. The open-label phase III study plans to enrol 200 participants, with an estimated primary completion date of July 2022.

IMCgp100 is an investigational drug that can refocus a T cell against the gp100 protein in uveal melanoma cells. IMCgp100 is a bispecific biologic with two functional ends, one targeting the soluble affinity enhanced T cell receptor (TCR) and the other an anti-CD3 single-chain variable fragment (scFv). The glycoprotein 100 (gp100) is presented in the context of HLA-A02, thus eligible patients must be HLA-A*02 positive. Two phase I trials have evaluated safety, pharmacokinetics, pharmacodynamics and efficacy for IMCgp100, which was administered weekly in HLA-A2 patients. The first-in-human (FIH) study enrolled 84 patients total with advanced melanoma including a cohort with UM (*n* = 16) (NCT01211262) [168]. The second study enrolled 19 patients with advanced UM using an intra-patient dose escalation (IE) regimen (four dose levels ranging from 54 to 73 mcg), designed to reduce T cell-mediated toxicities that were previously reported (NCT02570308). In both of the tow study durable and objective responses were noted. Rash, pruritus and peripheral edema were the most frequent related AEs observed. Hypotension noted with introductory doses was consistent with chemokine release and movement of lymphocytes into tissues. Notably, immunofluorescence studies reveal an infiltration of PD-1+/CD8+ T cells in tumour specimens with PD-L1 expression, confirming immune response activation within 24 h of the initial treatment dose. Preliminary results of the phase II study using the intra-patient escalation dosing regimen were recently presented at the 2018 Association for Research in Vision and Ophthalmology (ARVO) Annual Meeting [168,169,170]. The 68-mcg dose was identified to be the maximum tolerated dose. The most common all-grade treatment-emergent adverse events (TEAEs) were pruritus (90%), pyrexia (84%), fatigue (84%), hypotension (74%), chills (63%), nausea (68%), dry skin (63%) and peripheral edema (63%). The most frequent grade 3/4 adverse events were fatigue (16%), hypotension (16%), erythema (16%) and macular rash (11%). The phase II portion of this study is now recruiting with a plan to achieve a cohort of 150 patients. The estimated completion date for this expansion trial is April 2019 (NCT02570308). In addition, another pivotal phase II trial is currently enrolling treatment-naïve patients with uveal melanoma to compare IMCgp100 against investigator’s choice of systemic therapy, including dacarbazine or the immunotherapies pembrolizumab or ipilimumab. The estimated primary completion date is July 2020 with the hope of enrolling more than 300 participants (NCT03070392).

Recently, adoptive T-cell therapy has shown favourable responses in multiple refractory solid tumours. Subset of UM tumour infiltrating lymphocytes (TILs) exhibited a robust level of reactivity. Based on these findings, a phase II study (NCT01814046) was conducted to determine if TILs could induce regression of metastatic uveal melanoma [171]. A total of 24 patients were enrolled and divided into two cohorts. One cohort of 22 patients received lymphodepleting conditioning chemotherapy of cyclophosphamide and fludarabine followed by a single intravenous infusion of autologous TILs and high-dose aldesleukin. The other cohort of two patients received the same regimen with no aldesleukin. Of the 22 evaluated patients, about 36% had objective tumour regression, such as seven partial responses (31.81%) and one complete response (4.54%). Common grade 3/4 adverse effects were related to the lymphodepleting preparative regimen. All patients experienced lymphopenia, neutropenia and thrombocytopenia, and in six patients (27.27%) an infection was documented [171,172].

Glembatumumab vedotin, a new antibody-drug conjugate, is also being investigated in a phase II study (NCT02363283) [173]. The accrual of 37 patients with metastatic uveal melanoma has recently been completed. Glembatumumab vedotin is a monoclonal antibody-drug conjugate directed at the glycoprotein NBM and linked to the cytotoxic microtubule inhibitor monomethyl auristatin E (MMAE), which combines an antibody directed at the transmembrane glycoprotein NMB (gpNMB) with the microtubule inhibitor monomethyl auristatin (MMAE). A preclinical study has demonstrated a high frequency of gpNMB expression in uveal melanoma and modest clinical activity has been demonstrated in patients with cutaneous melanoma. Thus, the rationale to explore antitumour activity in patients with uveal melanoma [174,175].

### 6.8. Epigenetic Approaches

In the past decade, an important role has been attributed to epigenetic mechanisms in the development of many pathologies, and new treatments interfering with epigenetic regulation have begun to appear. As mentioned above, alterations in tumour suppressor genes or oncogenes can be generated by mutations or by transcriptional regulation by epigenetic mechanisms. These include DNA methylation or demethylation and/or histone acetylation or deacetylation. Notably, recent studies have found that changes in microRNAs and long ncRNAs play a role in the development and metastasis of UM [176,177,178,179,180]. A relatively new class of anti-cancer agents has been developed: histone deacetylase (HDAC) inhibitors. These agents have been demonstrated to induce histone and protein acetylation, and to alter gene expression, inducing death, apoptosis and cell cycle arrest in cancer cells [178,181]. Vorinostat is an orally bioavailable inhibitor of class I and II HDACs. Vorinostat is FDA approved for the management of cutaneous T cell lymphoma. It is being investigated both as mono-therapy and combination therapy for other types of cancers, including metastatic UM. A phase 2 study of Vorinostat (NCT01587352) in metastatic UM has terminated its enrolment, but results are yet to be published. Vorinostat is currently being evaluated in adjuvant and metastatic settings, respectively (NCT02068586, NCT01587352).

The cancer-testis antigen, a preferentially expressed antigen in melanoma (PRAME) is an independent prognostic biomarker in UM, which identifies increased metastatic risk in patients with Class 1 or disomy 3 tumour [75]. It has been considered another therapeutic target based on its lack of expression in normal cells [76]. Furthermore, in an experimental study using a retrospective cohort of 64 patients, Gezgin et al. [182] stated that 69% of metastatic uveal melanoma tumours analysed expressed PRAME. Additionally, the authors observed a concomitant expression of PRAME and HLA class I in metastases, suggesting a potential role for treatment with PRAME-directed immunotherapy. A Phase I/II study is evaluating the safety and activity of rimiducid, a genetically-modified autologous T cell product incorporating an HLA-A2-restricted PRAME-directed TCR, in patients with relapsed acute myeloid leukaemia, previously treated for myelodysplastic syndrome, and metastatic uveal melanoma (NCT02743611).

## 7. Conclusions

UM is a rare but aggressive tumour of the eye. Despite the great progress in its clinical management, UM therapy requires specific new approaches. Thus, UM needs additional investigations that aim at identify novel therapeutic strategies, which will lead to improved patient outcomes. To this end, a combination of different therapeutic approaches seem to open new roads in UM clinical management. Next investigations should be addressed to better understand the role of different altered genes/dysregulated pathways in the UM onset/progression. The new discoveries in the field will allow for the improvement of UM treatments/therapies.

## Figures and Tables

**Table 1:** Main gene mutations in uveal melanoma.

Gene Mutation	Gene Function	Dysregulation	Reference
*GNAQ/GNA11*	Responsible for extracellular signal transduction and intracellular pathways activation	91% of UM patients (principal driver of carcinogenesis) *GNAQ*/*GNA11* mutations are mutually exclusive Mutations in *GNAQ* have a prevalence of 42.2%, whereas in *GNA11* is 32.6%. *GNAQ*/*GNA11* mutations activate MEK and the oncoprotein YAP	[31,32,33,34,35,36,37,38]
*CYSLTR2*	Involved in leukotriene-mediated signalling in inflammation and fibrosis	Leu129Gln substitution founded only in samples lacking mutations in *GNAQ*, *GNA11*, and *PLCB4* activation of Gα_q_ signalling	[39,40]
*PLCB4*	Roles in the metabolism of inositol lipids and cancer	*PLCB4* p.D630Y mutations are mutually exclusive with mutations in *GNA11* and *GNAQ*	[41]
*BAP1*	Nuclear ubiquitin carboxyl-terminal hydrolase enzyme with deubiquitinase activity	22% in familial UM (associated with early UM onset) *BAP1* mutations cause loss of protein expression and loss of function and cell phenotype modifications *BAP1* depletion increases the amount of transmigration in UM cells	[23,39,40,41,42,43,44,45,46,47,48,49]
*SF3B1*	Involved in mRNA splicing	Detected in 15%–19% of UM cases Mutation at codon 625 of *SF3B1* gene is associated with UM development. Cause splicing dysregulations and alter the transcription process Mutually exclusive to *BAP1* mutations Associated with the chromosome 3 disomy Associated with good prognosis and low metastatic potential	[54,55,56,57]
*EIF1AX*	Stimulates the transfer of Met-tRNAi to the ribosomal subunit	Detected in 8%–18.9% of primary UM cases. Alterations in *EIF1AX* gene are usually missense mutations It was suggested that an abnormal translation process might be responsible for a clonal selective advantage in cells displaying this alteration	[29,32,58,59]
*TERT*	Catalytic subunit of the enzyme telomerase	It is present in about 1% of UM cases, whereas it is more common in other cancers *TERT* promoter mutations lead to the immortalization of somatic cells. Tumours carrying this mutation also showed *GNA11* gene alteration.	[32,60]

*GNAQ* (G protein subunit alpha q), *GNA11* (G protein subunit alpha 11), *CYSLTR2* (cysteinyl leukotriene receptor 2), *PLCB4* (phospholipase C, β4), *BAP1* (BRCA1-associated protein 1), *SF3B1* (splicing factor 3B subunit 1), *SRSF2* (serine and arginine rich splicing factor 2), *EIF1AX* (X-linked eukaryotic translation initiation factor 1A) and *TERT* (telomerase reverse transcriptase.
